# Normal Performance of *Fmr1* Mice on a Touchscreen Delayed Nonmatching to Position Working Memory Task^1^,^2^,^3^

**DOI:** 10.1523/ENEURO.0143-15.2016

**Published:** 2016-03-15

**Authors:** Prescott T. Leach, Jane Hayes, Michael Pride, Jill L. Silverman, Jacqueline N. Crawley

**Affiliations:** Department of Psychiatry and Behavioral Sciences, MIND Institute, University of California, Davis, School of Medicine, Sacramento, California 95817

**Keywords:** cognition, fragile X, learning and memory, mouse model, touchscreen, working memory

## Abstract

Fragile X syndrome is a neurodevelopmental disorder characterized by mild-to-severe cognitive deficits. The complete absence of *Fmr1* and its protein product in the mouse model of fragile X (*Fmr1* KO) provides construct validity. A major conundrum in the field is the remarkably normal performance of *Fmr1* mice on cognitive tests in most reports. One explanation may be insufficiently challenging cognitive testing procedures. Here we developed a delayed nonmatching to position touchscreen task to test the hypothesis that paradigms placing demands on working memory would reveal robust and replicable cognitive deficits in the *Fmr1* KO mouse. We first tested *Fmr1* KO mice (*Fmr1*) and their wild-type (WT) littermates in a simple visual discrimination task, followed by assessment of reversal learning. We then tested *Fmr1* and WT mice in a new touchscreen nonmatch to position task and subsequently challenged their working memory abilities by adding delays, representing a higher cognitive load. The performance by *Fmr1* KO mice was equal to WTs on both touchscreen tasks. Last, we replicated previous reports of normal performance by *Fmr1* mice on Morris water maze spatial navigation and reversal. These results indicate that, while the *Fmr1* mouse model effectively recapitulates many molecular and cellular aspects of fragile X syndrome, the cognitive profile of *Fmr1* mice generally does not recapitulate the primary cognitive deficits in the human syndrome, even when diverse and challenging tasks are imposed.

## Significance Statement

Traditional cognitive tests have revealed surprisingly normal performance in the *Fmr1* knock-out mouse model (*Fmr1*) of fragile X syndrome (FXS). Here we introduce novel methods for conducting working memory tasks, following discrimination and reversal learning tasks, to interrogate *Fmr1* mice with a diverse set of cognitive challenges. Touchscreen technology, incorporating direct analogies to methods used to evaluate cognitive abilities in human subjects and nonhuman primates, was used to evaluate *Fmr1* mice on simple pairwise discrimination, reversal, nonmatching to position, and delayed nonmatching to position. No significant deficits were detected in *Fmr1* mice, supporting the interpretation that this widely used mouse model of FXS is not optimal for discovering pharmacological therapeutics that improve cognitive functioning in individuals with FXS.

## Introduction

Fragile X syndrome (FXS) is a genetic disorder caused by a hypermethylated *FMR1* gene, which reduces expression of fragile X mental retardation protein (FMRP; Sutcliffe et al., 1992; Feng et al., 1995). FXS confers intellectual disability in domains such as working memory, executive function, short-term visual memory, visuospatial processing, sequence processing, and attention (Cianchetti et al., 1991; Freund and Reiss, 1991; Maes et al., 1994; Kogan et al., 2009; Baker et al., 2011). To understand the biological consequences of the absence of FMRP, The Dutch-Belgian Fragile X Consortium (1994) generated the *Fmr1* knock-out (KO) mouse (*Fmr1*) in 1994. This genetic mouse model of FXS has been extensively used to investigate the functional outcomes of loss of Fmr1. Surprisingly, cognitive deficits in *Fmr1* mice have proven remarkably mild and somewhat inconsistent across publications (Kooy, 2003). Background strain (Paradee et al., 1999; Dobkin et al., 2000; Spencer et al., 2011) and testing protocol differences across laboratories may explain the lack of well replicated learning and memory impairments in *Fmr1* mice in some cases. However, given the primary symptom of intellectual disability in humans with FXS, cognitive deficits in *Fmr1* KO mice were expected to be robust enough to withstand some variability in methods, background genetics, and environmental issues. It is important to know whether the results from the *Fmr1* mouse are informative for the development of treatments for FXS or whether other models would allow greater predictive validity.

Recently, touchscreen behavioral testing equipment ideal for evaluating complex learning and memory in rodents was introduced by Bussey et al. (2001) at the University of Cambridge and rigorously validated in mice by Brigman and Rothblat (2008) and others (Bussey et al., 2012; Mar et al., 2013; Oomen et al., 2013; Graybeal et al., 2014). Visually based, touch-sensitive technology in operant chambers is similar to cognitive testing designs in higher-order species, including nonhuman primates and humans (Green et al., 2009; Van der Molen et al., 2010; van Nieuwpoort et al., 2011; Berry-Kravis et al., 2013; Díez-Juan et al., 2014). Investigations using mouse touchscreen chambers have recently been published by several behavioral neuroscience laboratories (Brigman et al., 2005; Talpos et al., 2010; Graybeal et al., 2011, 2014; Romberg et al., 2011; Bussey et al., 2012; Silverman et al., 2015). The touchscreen technology offers the possibility of designing cognitive tasks with increasing difficulty to challenge specific cognitive domains affected by neurodevelopmental disorders, such as working memory. Working memory tasks, such as delayed nonmatching to position can be conducted in rodents using automated operant technology (Estapé and Steckler, 2002; Martin et al., 2004; Dowdy-Sanders and Wenger, 2006; Krueger et al., 2006, 2009; Bernardo et al., 2007; Goto et al., 2010a,b; Whitney and Wenger, 2012). The few reports of working memory in *Fmr1* mice have used radial arm maze, reporting mild reference memory deficits on the first 3 training days (Yan et al., 2004), and Morris water maze serial reversal learning, reporting reversal deficits (Baker et al., 2010). Touchscreen-based tasks, in which the mouse performs more naturalistic touching of the nose to the front panel instead of performing a lever press, has begun to be applied to *Fmr1* mice (Dickson et al., 2013).

We hypothesized that challenging touchscreen paradigms of working memory would detect cognitive deficits in *Fmr1* mice that were not detectable with arguably simpler tasks in the literature. The present experiments were designed with the following two goals in mind: (1) to optimize parallels with human FXS testing equipment, such as the Cambridge Neuropsychological Test Automated Battery (CANTAB), which has been successfully used in subjects with intellectual disability (Green et al., 2009; van Nieuwpoort et al., 2011) including FXS (Van der Molen et al., 2010; Berry-Kravis et al., 2013); and (2) to identify cognitive deficits in tasks that include the capacity to increase demands on working memory. Increasing the working memory load by introducing long delays was designed to determine whether the normal performance of *Fmr1* mice on the tasks previously used may have been due to the insufficiently challenging nature of standard learning and memory tasks commonly used in mice or the specific cognitive modalities used in those tasks. Further, a comparison of recognition memory, working memory, and spatial navigation with the Morris water maze could reveal novel insights as to the cognitive profile of *Fmr1* KO mice. Analogous CANTAB testing in humans with FXS revealed mean mental ages ranging from 4.53 ± 0.59 to 7.38 ± 2.83 years compared with chronological ages ranging from 25.65 ± 7.41 to 30.13 ± 8.97 years on visuospatial short term memory (sequential), working memory (self-ordered search), spatial recognition memory, recognition memory (instant recall), and recognition memory (delayed recall; Van der Molen et al., 2010). Strong cognitive deficits in an *Fmr1* mouse model in a task with face validity to touchscreen methods used in humans with FXS would offer a novel preclinical research tool to test compounds for therapeutic efficacy.

## Materials and Methods

### Subjects

Breeding pairs of *Fmr1* knock-out mice (*Fmr1*) on the FVB Pde6b+ Tyrc-ch/AntJ background with normal vision (catalog #004624), the background inbred strain FVB Pde6b+ Tyrc-ch/AntJ (FVB/AntJ; catalog #004828) with normal vision, and male C57BL/6J (B6) mice (catalog #000664) were purchased from The Jackson Laboratory. Mice were bred and maintained in an AAALAC-accredited vivarium on a conventional lighting schedule, with temperature and humidity controls. Offspring were weaned at 21 d of age, and were housed with littermates by sex in mixed-genotype cages, with two to four mice per cage. *Fmr1* and their wild-type (WT) littermates were maintained on the FVB/AntJ background. The breeding scheme was *Fmr1* heterozygous females × WT males. Genotyping was conducted using a Bio-Rad thermocycler and REDExtract-N-Amp PCR ReadyMix (catalog #R4775, Sigma-Aldrich) with primers targeting the WT (TGT GAT AGA ATA TGC AGC ATG TGA), mutated (CAC GAG ACT AGT GAG ACG TG), and common primer sequences (CTT CTG GCA CCT CCA GCT T). Genotypes were recorded in a notebook with corresponding subject mouse identification (ID) numbers. At weaning, each mouse was assigned a unique cage card ID number that did not include mouse ID numbers or genotype. Experimenters were aware of only the unique cage card ID number when testing mice, thereby preventing bias by the researcher.

Male *Fmr1* (y/−) and male WT littermates (y/+) were used for the touchscreen and water maze experiments. Control experiments were conducted with the inbred strains B6 and FVB/AntJ. Behavioral testing was conducted during the light phase, between 9:00 A.M. and 5:00 P.M. Prior to touchscreen testing and during water maze testing, mice were maintained in the same postweaning cages, in the same vivarium, and were allowed *ad libitum* access to food and water. Food restriction for the touchscreen experiments was initiated at 8-16 weeks of age, beginning ∼1 week before the start of habituation. Eighty-five to 90% of free-feeding body weight was maintained throughout the touchscreen testing period. All procedures were approved by the University of California, Davis, Institutional Animal Care and Use Committee, and followed the National Institutes of Health *Guide for the Care and Use of Laboratory Animals*.

### Touchscreen apparatus

Bussey-Saksida touchscreen chambers and software, manufactured by Campden Instruments, were purchased from Lafayette Instruments. Boxes were trapezoidal to enhance the focus of attention on the front screen, fitted with a Plexiglas two-hole mask (pairwise discrimination) or a five-hole mask (nonmatch). Screen covers contained 4 × 4 cm openings (five-hole mask) or 8 × 8 cm openings (two-hole mask) in which visual images were projected. Each touchscreen box contained a peristaltic pump that delivered a liquid reinforcement of 20 µl of Ensure strawberry milkshake, diluted 1:1 with distilled H_2_O, into a food magazine located on the back of the touchscreen chamber. Mice were loaded into the chambers based on their unique cage card ID numbers, and software automatically ran the program and collected response data, thus preventing the introduction of any observer bias by the investigator.

**Figure 1.**
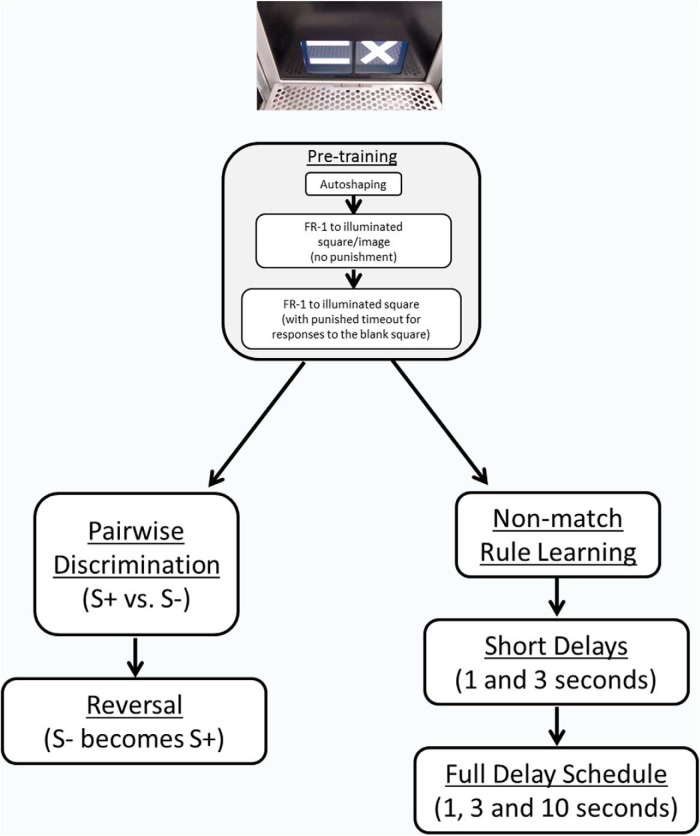
Diagram of touchscreen training and testing schedules. Top, An image of the touchscreen chamber is shown when configured for pairwise visual discrimination. The stages of training for pairwise visual discrimination (left) and delayed nonmatch to position (right) are shown, with similar pretraining shown in the middle. Pretraining for both tasks consists of autoshaping to the food magazine, FR-1 to the illuminated square/image (without punishment for presses to the blank space), and FR-1 to the illuminated square/image (with a punished timeout period for presses to the blank space). Pairwise visual discrimination pretraining included the following two additional stages: (1) after autoshaping, mice received 1 d where they received reward either for an active screen touch, or after 30 s, whichever came first, and thereafter, all trials were “forced trials”; and (2) before punishment was added for blank responses, mice had to “initiate” trials with a nosepoke into the food magazine. Subsequently, pairwise visual discrimination consists of the discrimination between S+ and S−, followed by reversal of reward contingencies. The images used in the present study are shown (above). For the nonmatch task, after abbreviated pretraining, mice first learned the nonmatch rule without delays, followed by short delays (1 and 3 s), and then were tested for 25 d at the full delay schedule (1, 3, and 10 s delays).

The testing sequence is illustrated in Figure 1, and described below.

### Pairwise visual discrimination

#### Pretraining

Each subject mouse was habituated to the touchscreen boxes prior to operant training. During habituation, the food magazine was initially filled with reinforcer and signaled with a small LED located directly above the food magazine. Each nosepoke in the food magazine initiated a new habituation trial, which consisted of a 10 s intertrial interval (ITI) and delivery of the 20 µl of reinforcer. Mice were habituated until 100 trials were reached in a 1 h period or until a clear pattern of increasing trials was observed for 3 d. Following habituation, mice received fixed-ratio (FR-1) training, where each touch to a randomly presented visual image on the screen was reinforced and paired with a brief auditory cue that acts as a conditioned stimulus (CS). Image location varied randomly between the left and right screen locations. The first day of FR-1 training had no scheduled contingencies for touching the blank image location. Trials resulted in presentation of a reinforcer either upon touching the image or after 30 s, whichever came first. Subsequently, mice received FR-1 training, which removed the 30 s contingency for automatic reinforcer presentation, such that only touches to the image resulted in the presentation of a reinforcer. Mice remained on this phase until they had received 30 reinforcers in a single session. The third phase of FR-1 incorporated the contingency of a food magazine entry to initiate each trial. Mice remained on this phase until they had received 30 reinforcers in a single session. The fourth phase of FR-1 added a 10 s punished timeout in the event of a blank image touch. An ITI of 20 s followed both correct and blank image touches. Software automatically calculated the percentage correct performance scores as #Image touches ÷ #Image touches + #blank touches. Training continued for 2 d.

#### Pairwise discrimination acquisition

Mice were trained in pairwise visual discrimination using methods based on previous seminal publications (Bussey et al., 2001; Brigman et al., 2008, 2009; Graybeal et al., 2014; Silverman et al., 2015). Pairwise visual discrimination training trials had two distinct images randomly presented in the image locations. Mice were assigned to be reinforced for touches to the correct screen image (S+) and punished with a 10 s timeout for touches to the other image (S−). The two images were an X or an =, which were matched for illumination and pseudorandomly assigned to individual mice as they finished pretraining. Approximately half of each genotype were assigned to X (four WT and four *Fmr1* mice), and the others were assigned to = (4 WT and 3 *Fmr1*) as the initial reinforced stimulus. Incorrect trials led to correction trials, which were identical to the previous trial in all ways. Responses on the correction trials were not included in the performance score calculations. Mice were trained until a performance criterion of ≥80% was reached for 2 days.

#### Pairwise discrimination reversal

After completing the acquisition phase of pairwise visual discrimination, the contingencies on the images were reversed. S+ became S−, and vice versa. Mice were trained on reversal until a performance criterion of ≥80% was reached for 2 days.

### Delayed nonmatch to position

#### Pretraining

Mice received 1 d of habituation to the touchscreen box. During habituation, the food magazine was initially filled with reinforcer and signaled with a small LED located directly above the food magazine. Each nosepoke in the food magazine initiated a new habituation trial, which consisted of a 10 s ITI and delivery of 20 µl of reinforcer. Following habituation, mice received FR-1 training, where each touch to a visual image on the screen was reinforced and paired with a brief auditory cue that acts as a CS. All images used were a filled square symbol at 100% illumination. Image location varied randomly between the far left and far right screen locations. The first day of FR-1 training had no punishment contingencies for touching blank screen locations, where no image was displayed. Subsequent touches to blank screen locations were punished by a 20 s timeout. An ITI of 20 s followed both correct and incorrect trials. After each session, the numbers of reinforced and nonreinforced touches were recorded and used to calculate the percentage correct performance scores, as follows: #Correct touches ÷ #Correct touches + #blank touches. Training continued until a performance criterion of ≥80% was reached on day 2.

#### Nonmatch training

The nonmatch contingency was introduced for each subject mouse when criterion was reached on the previous phase. Briefly, a sample image was presented, as in the previous phase, but a touch to the sample image triggered the activation of the reward magazine light and auditory CS. In this phase, a nosepoke in the food magazine initiated a choice between images in the two active spatial locations, (far left and far right). During the choice part of the trial, touches to the image in the other spatial location (i.e., nonmatches) were reinforced, while touches to the previous image location (i.e., matches) were punished with a 20 s timeout. Training continued until a performance criterion of ≥80% was reached for 2 d.

#### Initial delay training

Next, 1 and 3 s mandatory delays were randomly assigned and imposed, after the sample image was pressed and before a food magazine entry initiated a choice. Training continued until a performance criterion of ≥80% at the more challenging 3 s delay was reached for 2 d.

#### Full delay testing

The complete set of nonmatch testing instituted 1, 3, and 10 s mandatory delays, randomly assigned, after the sample image was pressed and before a food magazine entry initiated a choice. Training continued for 25 d for all mice (see Movie 1 for video clip of successful full delay testing).

Movie 1. Performance in the touchscreen apparatus on delayed nonmatching to position in a representative FVB/AntJ mouse. Two trials are shown, and the sample appears in the left location for both. After touching the sample image, the mouse turns to the back of the chamber to nose poke in the reward tray. After a random delay (1, 3, or 10 s), the nose poke initiates a choice (match and nonmatch) where the image appears on both sides of the touchscreen. For both trials shown, the mouse correctly nonmatches and earns a reward. 10.1523/ENEURO.0143-15.2016.video.1

### Morris water maze

To complement the touchscreen assays, we used the standard Morris water maze task to evaluate hippocampal-dependent spatial navigation learning and memory in *Fmr1* mice (Morris et al., 1982; Moser et al., 1993; Logue et al., 1997). The water maze was a 120 cm circular pool, filled 45 cm deep with 24°C water made opaque with nontoxic white paint (Crayola) containing a 12 cm platform located 1 cm beneath the water. External cues to aid spatial navigation included a prominent sink, computer, water temperature regulator with hose, a large black X on the wall, and a yellow paper lantern hung from the ceiling. Trials were video recorded and scored by automated software (EthoVision, Noldus) for measures including latency to find the hidden platform, total distance traveled, and swim speed. Mice were trained in the hidden platform version of the Morris water maze in a manner consistent with methods that are standard in the literature (Zeng et al., 2001; Bourtchouladze et al., 2006; Daumas et al., 2008; Yang et al., 2012). Since previous literature on Morris water maze performance by *Fmr1* mice has included normal performance in some reports and impaired performance in other reports (The Dutch-Belgian Fragile X Consortium, 1994; Kooy et al., 1996; D'Hooge et al., 1997; Paradee et al., 1999; Yan et al., 2004; Baker et al., 2010; Uutela et al., 2012; Tian et al., 2015), we chose to modify the standard procedure slightly to make it more challenging by reducing the number of daily training trials from four to three. Briefly, each *Fmr1* or WT mouse was placed into the water maze, facing the wall, in one of four possible quadrant locations, which differed pseudorandomly by training day. Mice were given 60 s to find the hidden platform. If a subject mouse was unable to find the platform by the end of 60 s, it was gently guided to the platform and allowed to rest for ∼10 s between trials. The hidden platform was in the same location, in the same quadrant, on each training day. Trials were given sequentially, with an ∼10 s platform rest interval. Mice were placed under infrared heating lamps after the last trial each day to prevent hypothermia. Acquisition was assessed daily until the WT group reached a latency criterion of <15 s to reach the hidden platform. Approximately 3 h after the last training trial, the platform was removed and mice underwent a 60 s probe trial to determine the amount of time spent exploring the target quadrant and the number of times the animal crossed the previous platform location and corresponding pseudoplatform locations in each quadrant. Since both genotypes reached criterion on the initial acquisition, reversal learning was then conducted with the platform in the opposite quadrant 1 d following the completion of acquisition. Reversal was conducted with methods identical to acquisition, with the new platform location being the only difference. Reversal was assessed daily until the WT group reached a latency criterion of <15 s to reach the hidden platform. The reversal probe trial was conducted ∼3 h after the last training trial.

### Statistical analyses

For pairwise visual discrimination and reversal touchscreen experiments, mixed-model 2 × 2 ANOVAs with genotype as a between-subjects factor and phase (acquisition or reversal) as within-subjects factors were conducted for days, and trials needed to reach criterion. One WT and two *Fmr1* mice did not reach the criterion for reversal at the end of study and were removed from the ANOVAs. Additionally, the days to criterion for each genotype were compared using Mantel–Cox (log-rank) survival curve analyses separately for acquisition and reversal. For the Mantel–Cox analysis, the three mice removed from the ANOVA analyses were included as censored subjects (i.e., these animals were used to calculate the proportion reaching criterion when their data were present but were not used to calculate this number when their data were absent). Analysis of delayed nonmatch to position (DNMTP) performance with 1, 3, and 10 s delays used a 3 × 25 within-subjects repeated-measures ANOVA with delay (1, 3, and 10) and day (1-25) as within-subjects factors. Repeated-measures ANOVA was conducted separately for each strain and genotype. Simple main effect tests on delays were conducted to confirm delay-dependent performance using Tukey’s *post hoc* tests to determine which delays were significantly different from each other. *Post hoc* tests were conducted using Bonferroni correction for multiple comparisons to determine significant differences between 1 and 3 s delays, and between 1 and 10 s delays, for each training day. Additionally, in order to directly compare strain and genotype performance, we conducted a mixed-model ANOVA with genotype or strain as between-subjects factors and delay as within-subjects factors. For nonmatch to position and early delayed nonmatch to position learning, when testing was limited to 1 and 3 s delays, mixed-model 2 × 2 ANOVAs with genotype (WT or KO) as a between-subjects factor and phase (nonmatch learning or initial delay acquisition) as within-subjects factors were conducted for days, and trials needed to reach criterion, where normality assumptions were satisfied. For nonmatch to position and early delayed nonmatch to position learning, the days to criterion were also compared using Mantel–Cox survival curve analyses.

For Morris water maze acquisition, mixed-model 2 × 8 ANOVAs with genotype as a between-subjects factor and training day as a within-subjects factor were conducted for latency to find the hidden platform (in seconds), total distance traveled (in centimeters), and swim speed (in centimeters per second). Repeated-measures ANOVAs were conducted for the probe trial performance on the time spent in each quadrant, and for the number of platform crossings in each quadrant, to determine whether each genotype had used distal spatial cues to locate the hidden platform during learning. For Morris water maze reversal, the same analyses were conducted except the mixed-model ANOVAs were 2 × 4 with genotype as a between-subjects factor and the 4 training days as a within-subjects factor. In the event of violations of sphericity, Greenhouse–Geisser corrections were used to compute the adjusted degrees of freedom and *p* values. For all significant probe trial ANOVAs, Dunnett’s *post hoc* tests were used to compare quadrant time and platform crossings for target quadrant versus nontarget quadrants, and for previous platform location versus pseudoplatform locations in each quadrant.

## Results

Complete statistical results for each experiment appear in Tables 1-5.

**Table 1:** Statistical results for pairwise visual discrimination acquisition and reversal in *Fmr1* and WT mice

Effect	Data structure	Type of test	Power	df (between)	df (within)	*F*	χ2	*p*
Pairwise discrimination phase (d)	Normally distributed	Two-factor repeated-measures ANOVA	0.96	1	9	17.58		0.002
Pairwise discrimination genotype (d)	Normally distributed	Two-factor repeated-measures ANOVA	0.41	1	9	3.78		0.08
Pairwise discrimination interaction (d)	Normally distributed	Two-factor repeated-measures ANOVA with *post hoc* Bonferroni correction	0.13	1	9	0.83		0.4
Pairwise discrimination phase (trials)	Normally distributed	Two-factor repeated-measures ANOVA	0.62	1	9	6.45		0.03
Pairwise discrimination genotype (trials)	Normally distributed	Two-factor repeated-measures ANOVA	0.16	1	9	1.10		0.3
Pairwise discrimination interaction (trials)	Normally distributed	Two-factor repeated-measures ANOVA	0.06	1	9	0.13		0.7
Pairwise discrimination (survival curve)	Normally distributed	Mantel–Cox test	0.06	1			0.53	0.5
Pairwise discrimination reversal (survival curve)	Normally distributed	Mantel–Cox test	0.07	1			0.48	0.5

### Performance of *Fmr1* and WT mice on pairwise discrimination and reversal learning

**Figure 2.**
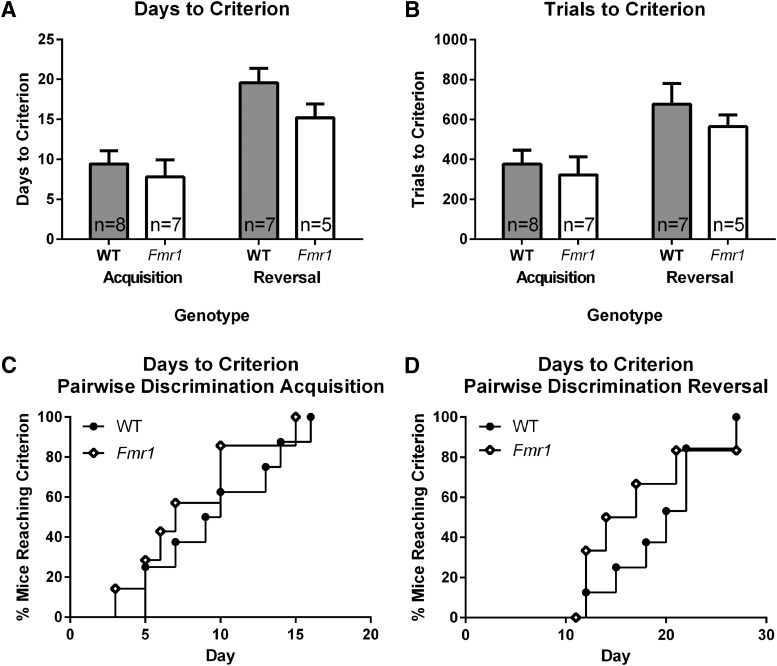
Pairwise visual discrimination showed no genotype differences in performance between *Fmr1* and WT mice. ***A***, Days to criterion for acquisition and reversal of mice completing both phases. ***B***, Trials to criterion for acquisition and reversal of mice completing both phases. ***C***, Days to criterion for acquisition, indicating proportion of individuals that had completed training at each day (survival curve). ***D***, Days to criterion for reversal, indicating the proportion of individuals that had completed reversal at each training day (survival curve).

Both *Fmr1* WT and KO mice reached criterion in the touchscreen visual discrimination task and subsequent reversal task (Fig. 2, Table 1). Comparing the number of days required to reach criterion for each phase of pairwise discrimination revealed that there was no effect of genotype and no phase × genotype interaction (Fig. 2*A*). Similarly, comparing the number of trials required to reach criterion showed no effect of genotype and no phase × genotype interaction (Fig. 2*B*). The number of trials required to reach criterion was significantly higher for reversal than acquisition, as expected. The number of days to reach criterion (survival curve analysis) for acquisition did not differ between *Fmr1* and WT mice (Fig. 2*C*). Similarly, there were no genotype differences in the number of days required to reach criterion during reversal (Fig. 2*D*).

### FVB and B6 working memory performance in touchscreen nonmatching to position

**Figure 3.**
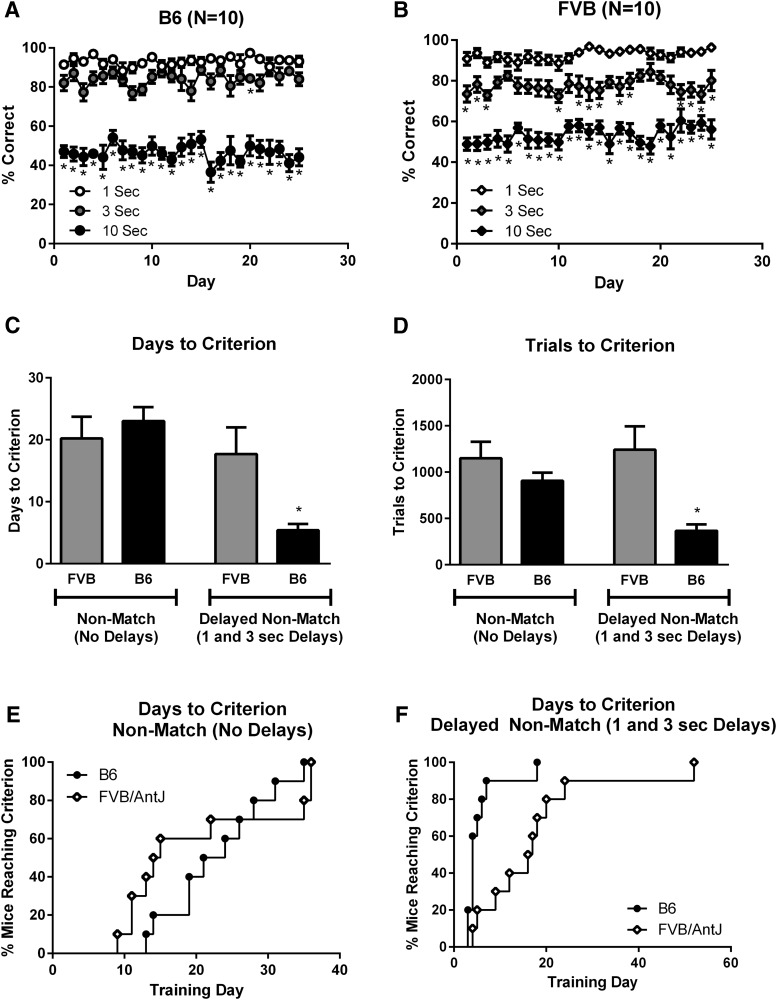
Validation of the delayed nonmatching to position task revealed superior working memory performance in B6 compared with FVB/AntJ inbred strains of mice. ***A***, B6 performance at 1, 3, and 10 s delays on the delayed nonmatching to position task. ***B***, FVB/AntJ performance at 1, 3, and 10 s delays. ***C***, ***D***, Days to criterion (***C***) and trials to criterion (***D***) for nonmatching to position rule learning (without delays), and acquisition of initial delayed nonmatching to position (1 and 3 s delays only). ***E***, Days to criterion (survival curves) for nonmatching rule acquisition, indicating the proportion of individuals that had completed training at each training day. ***F***, Days to criterion for the acquisition at 1 and 3 s delays, indicating the proportion of individuals that had completed this phase at each training day. *p < 0.05 compared with 1 s delay (***A***, ***B***); *p < 0.05, strain difference for all days and trials to criterion in ***C–F***.

Inbred strains were used to develop a touchscreen task that would challenge a different cognitive domain than simple pairwise visual discrimination. To this end, we adapted standard approaches for delayed nonmatching to position. Validation used two strains of mice, C57BL/6J (B6), which is frequently used as a genetic background for targeted mutations, and FVB Pde6b+ Tyrc-ch/AntJ (FVB/AntJ), the background strain for the *Fmr1* mice used in the present studies. A successfully validated working memory task should display delay-dependent performance without mediating strategies such as using body-positioning techniques to lower the working memory demand. Shaping and training required 20–85 d. Asymptotic delay–schedule performance (Fig. 3*A*,*B*) was conducted for 25 d. B6 (Fig. 3*A*) displayed delay-dependent performance, such that the percentage correct at various delays followed the expected order of working memory load (i.e., performing better at 1 > 3 > 10 s delays), confirmed with simple main-effect analyses (Table 2). FVB/AntJ (Fig. 3*B*) mice displayed delay-dependent performance in a similar fashion (i.e., 1 > 3 > 10 s). Comparing daily scores at each delay revealed similar performance at 1 and 3 s in B6 mice on 24 of the 25 testing days, indicating that the majority of the delay-dependent performance occurred at the 10 s delay. FVB/AntJ mice exhibited significantly better performance at 1 s than at 3 s on 13 of 25 d. The days required to reach criterion are graphed for illustrative purposes in Figure 3*C*. However, due to violations of normality, a traditional mixed-model ANOVA was not conducted for this parameter. Motivation was examined by analysis of the number of trials completed. A mixed-model ANOVA with strain as a between-subjects factor and training phase as a within-subjects factor revealed a significant effect of strain and a significant interaction. *Post hoc* analysis revealed a significant difference between genotypes on acquisition of the initial delays, indicating that B6 mice required fewer trials to reach criterion at the initial delays (1 and 3 s), although they required a similar number of trials for the initial acquisition of the nonmatch rule. As an additional method for calculating differences between strains on nonmatch learning and initial delay acquisition, and due to the violations of normality described above, days to criterion (survival) analyses were conducted to compare the rates at which each strain met the criterion of ≥80% performance for 2 d. Days to criterion analysis showed no strain differences between B6 and FVB/AntJ mice during nonmatch acquisition. During acquisition of the 1 and 3 s delays, B6 mice reached criterion significantly faster than FVB/AntJ mice. The median number of days to criterion for B6 was 4 d, compared with 16.5 d for FVB/AntJ mice. Finally, to directly compare performance at each delay across strains, we ran a mixed-model ANOVA with strain as a between-subjects factor and delay as a within-subjects factor. Direct comparison of B6 and FVB/AntJ mice revealed similar performance at a 1 s delay, but significant differences were observed at 3 and 10 s delays. B6 mice performed significantly better than FVB/AntJ mice at 3 s delay, but B6 mice performed significantly worse than FVB/AntJ mice at 10 s delay.

**Table 2:** Statistical results for nonmatching to position and delayed nonmatching to position performance in B6 and FVB mice

Effect	Data structure	Type of test	Power	df (between)	df (within)	*F*	χ^2^	*t*	*p*
B6 day (DNMTP)	Sphericity passed	Two-factor repeated-measures ANOVA	0.78	24	216	0.97			0.5
By delay (DNMTP)	Sphericity passed	Two-factor repeated-measures ANOVA	1.00	2	18	386.96			1.67E-15
B6 interaction (DNMTP)	Sphericity passed	Two-factorrepeated-measures ANOVA with *post hoc* Bonferroni correction	0.94	48	432	0.92			0.6
Simple effects test (DNMTP)	Normally distributed	One-way ANOVA with *post hoc* Bonferroni correction	1.00	2	18	387.00			1.67E-15
1 vs 3 s (DNMTP)	Normally distributed	*Post hoc* Bonferroni correction	1.00		9			4.99	0.0004
3 vs 10 s (DNMTP)	Normally distributed	*Post hoc* Bonferroni correction	1.00		9			21.21	0.00000002
FVB day (DNMTP)	Sphericity passed	Two-factor repeated-measures ANOVA	0.73	24	216	0.89			0.6
FVB delay (DNMTP)	Sphericity passed	Two-factor repeated-measures ANOVA	1.00	2	18	582.60			3.22E-15
FVB interaction (DNMTP)	Sphericity passed	Two-factor repeated-measures ANOVA with *post hoc* Bonferroni correction	0.98	48	432	1.10			0.3
Simple effects test (DNMTP)	Normally distributed	One-way ANOVA with *post hoc* Bonferroni correction	1.00	2	18	582.60			3.22E-15
1 vs 3 s	Normally distributed	*Post hoc* Bonferroni correction	1.00		9			13.02	0.0000002
3 vs 10 s	Normally distributed	*Post hoc* Bonferroni correction	1.00		9			20.81	0.000000007
Nonmatch acquisition (strain survival curve)	Not normally distributed	Mantel–Cox test	0.11	1			0.03		0.9
Initial delay acquisition (strain survival curve)	Not normally distributed	Mantel–Cox test	0.73	1			7.83		0.005
Nonmatch and delayed nonmatch acquisition (strain)	Normally distributed	Two-factor repeated-measures ANOVA	0.80	1	18	8.70			0.009
Nonmatch and delayed nonmatch acquisition (phase)	Normally distributed	Two-factor repeated-measures ANOVA	0.35	1	18	2.72			0.1
Nonmatch and delayed nonmatch acquisition (interaction)	Normally distributed	Two-factor repeated-measures ANOVA with *post hoc* Bonferroni correction	0.60	1	18	5.41			0.03
Strain comparison (strain)	Normally distributed	Mixed-model ANOVA	0.05	1	18	0.002			0.97
Strain comparison (delay)	Normally distributed	Mixed-model ANOVA	1.00	2	36	869.6			0.000000
Strain comparison (interaction)	Normally distributed	Mixed-model ANOVA	1.00	2	36	20.68			0.000001

#### Performance of *Fmr1* and WT mice in touchscreen delayed nonmatch to position task

**Figure 4.**
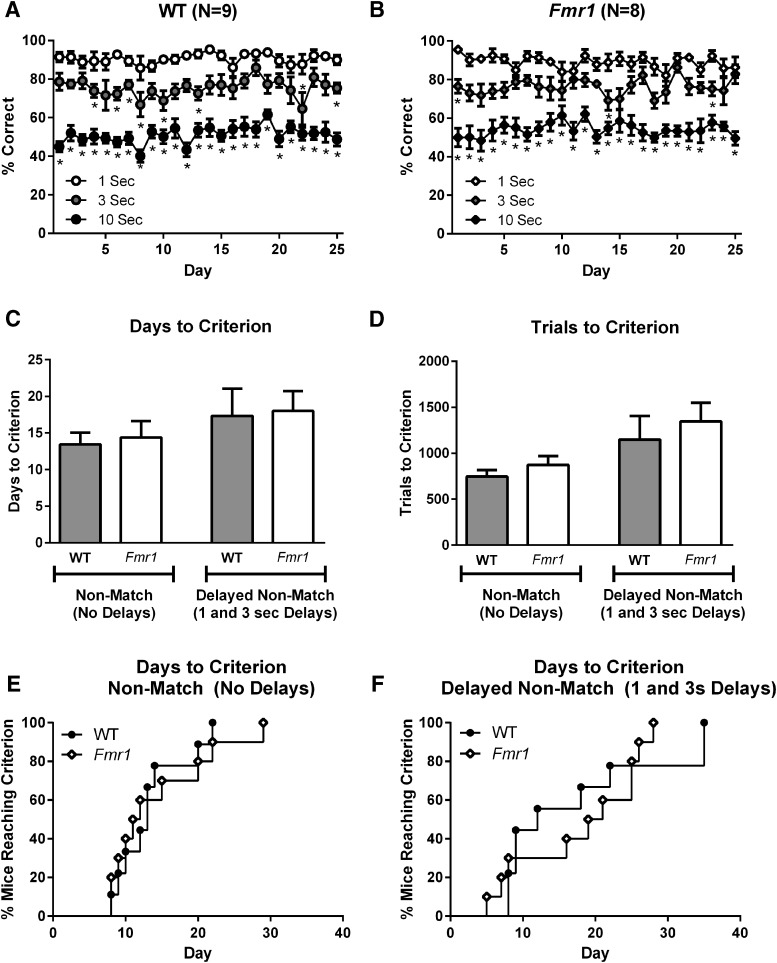
Delayed nonmatching to position showed no genotype differences between *Fmr1* and WT mice. ***A***, WT performance on delayed nonmatching to position at 1, 3, and 10 s delays. ***B***, *Fmr1* performance on delayed nonmatching to position at 1, 3, and 10 s delays. ***C***, ***D***, Days to criterion (***C***) and trials to criterion (***D***) for rule learning in nonmatching to position (without delays) and acquisition of the initial delay periods (1 and 3 s delays only). ***E***, Days to criterion for nonmatch rule acquisition, indicating the proportion of individuals that had completed training at each training day. ***F***, Days to criterion for short delay (1 and 3 s) acquisition, indicating the proportion of individuals that had completed this phase across time. *p < 0.05 indicates significant difference compared with 1 s delay on full schedule performance.

After successful validation of the touchscreen version of delayed nonmatching to position with the B6 and FVB/AntJ inbred strains, we proceeded to test the working memory capacity of a new cohort of *Fmr1* and WT mice. After extensive shaping and training, consistent and delay-dependent performance was seen over the 25 d of testing (Fig. 4, Table 3). Both WT and *Fmr1* mice displayed delay-dependent deficits, with better choice accuracies at 1 s than at 3 s, and better choice accuracies at 3 s than at 10 s. Comparing daily performance between the 1 s delay and each other delay revealed a significant difference between 1 and 3 s on 8 of 25 d for WT mice, and 3 of 25 d for *Fmr1* mice, between 1 and 10 s for WT mice on 24 of 25 d, and between 1 and 10 s for *Fmr1* mice on 20 of 25 d. The days to criterion (survival curve analyses) revealed that the performances of WT and *Fmr1* mice were similar on both nonmatch acquisition and delay acquisition. Two *Fmr1* mice exhibited spontaneous seizures in their home cages after completing days 5 and 7 of the final delay schedule. Scores from these two subject mice were removed from the final delay schedule statistics and graphs, but were retained in the acquisition dataset. Direct comparison of performance at each delay across genotype with a mixed-model ANOVA, as conducted above, revealed similar performances among genotypes at all delays.

**Table 3:** Statistical results for nonmatching to position and delayed nonmatching to position in *Fmr1* and WT mice

Effect	Data structure	Type of test	Power	df (between)	df (within)	*F*	χ^2^	*t*	*p*
WT day (DNMTP)	Sphericity passed	Two-factor repeated-measures ANOVA	0.92	24	192	1.34			0.1
WT delay (DNMTP)	Sphericity passed	Two-factor repeated-measures ANOVA	1.00	2	16	237.31			1.28E-12
WT interaction (DNMTP)	Sphericity passed	Two-factor repeated-measures ANOVA with *post hoc* Bonferroni correction	0.93	48	384	0.90			0.7
Simple effects test (DNMTP)	Normally distributed	One-way ANOVA with *post hoc* Bonferroni correction	1.00	2	16	237.30			1.28E-12
1 vs 3 s	Normally distributed	*Post hoc* Bonferroni correction	1.00		8			8.25	0.0002
3 vs 10 s	Normally distributed	*Post hoc* Bonferroni correction	1.00		8			13.34	0.000007
*FMR1* day (DNMTP)	Sphericity passed	Two-factor repeated-measures ANOVA	0.53	24	168	0.65			0.9
*FMR1* delay (DNMTP)	Sphericity passed	Two-factor repeated-measures ANOVA	1.00	2	14	162.69			0.0000000002
*FMR1* interaction (DNMTP)	Sphericity passed	Two-factor repeated-measures ANOVA with *post hoc* Bonferroni correction	0.96	48	336	1.01			0.5
Simple effects test (DNMTP)	Normally distributed	One-way ANOVA with *post hoc* Bonferroni correction	1.00	2	14	162.70			0.0000000002
1 vs 3 s	Normally distributed	*Post hoc* Bonferroni correction	1.00		7			6.57	0.0009
3 vs 10 s	Normally distributed	*Post hoc* Bonferroni correction	1.00		7			11.26	0.0000003
Nonmatch acquisition (genotype survival curve)	Not normally distributed	Mantel–Cox test	0.98	1			0.11		0.7
Initial delay acquisition (genotype survival curve)	Not normally distributed	Mantel–Cox test	0.95	1			0.13		0.7
Nonmatch and delayed nonmatch acquisition (genotype)	Normally distributed	Two-factor repeated-measures ANOVA	0.14	1	17	0.84			0.4
Nonmatch and delayed nonmatch acquisition (phase)	Normally distributed	Two-factor repeated-measures ANOVA	0.69	1	17	6.85			0.02
Nonmatch and delayed nonmatch acquisition (interaction)	Normally distributed	Two-factor repeated-measures ANOVA	0.05	1	17	0.05			0.8
Genotype comparison (genotype)	Normally distributed	Mixed-model ANOVA	0.09	1	15	0.4272			0.5
Genotype comparison (delay)	Normally distributed	Mixed-model ANOVA	1.00	2	30	393.9			0.000000
Genotype comparison (interaction)	Normally distributed	Mixed-model ANOVA	0.28	2	30	1.43			0.3

#### Performance of *Fmr1* and WT in Morris water maze acquisition

**Figure 5.**
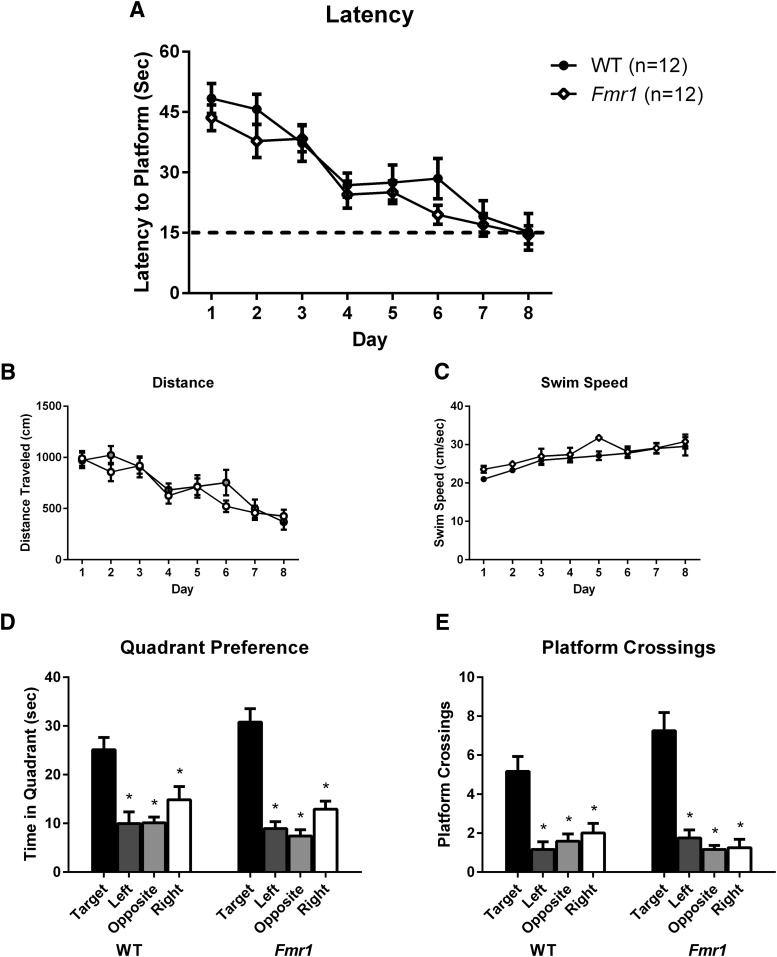
Acquisition of Morris water maze hidden platform spatial navigation learning showed no genotype differences between *Fmr1* and WT mice. Both genotypes displayed normal performance during acquisition. ***A***, Latency to find the hidden platform. ***B***, Distance traveled during the training trials. ***C***, Swim speed during the training trials. ***D***, Quadrant time during the 60 s probe trial, begun 3 h after the last training trial. ***E***, Platform location crossings during the 60 s probe trial. *p < 0.05 indicates more time in the previously trained platform quadrant than in the other three quadrants, and more crossings over the previous platform location than over the other three pseudolocations.

**Figure 6.**
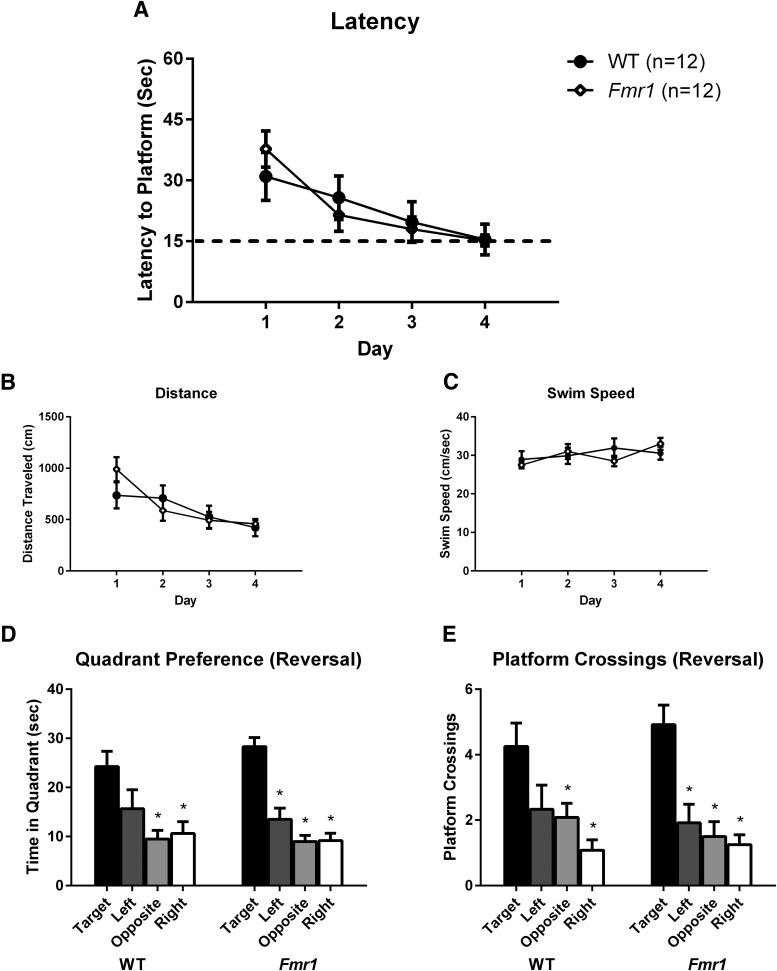
Reversal of Morris water maze hidden platform spatial navigation learning showed no genotype differences between *Fmr1* and WT mice. Both genotypes displayed normal performance during reversal. ***A***, Latency to find the hidden platform. ***B***, Distance traveled during the training trials. ***C***, Swim speed during the training trials. ***D***, Quadrant time during the 60 s probe trial, begun 3 h after the last training trial. ***E***, Platform location crossings during the 60 s probe trial. *p < 0.05 indicates more time in the previously trained platform quadrant than in the other three quadrants, or more crossings over the previous platform location than over the other three pseudolocations.

Both *Fmr1* and WT mice on the sighted FVB/AntJ inbred background strain performed normally on Morris water maze hidden platform learning (Fig. 5, Table 4). As expected, a significant effect of training day was seen (Fig. 5*A*). No effect of genotype and no day × genotype interaction were detected for latency measures. For distance traveled (Fig. 5*B*), a significant effect of training day was detected; with no effect of genotype and no day × genotype interaction. Swim speed (Fig. 5*C*) analysis revealed a significant effect of training day, no effect of genotype, and no day × genotype interaction. Probe trial performance 3 h after training on day 8 revealed significant quadrant preference (Fig. 5*D*) and selective target search (Fig. 5*E*) for both WT and *Fmr1* mice, supporting the interpretation that the hidden platform task was learned using distal environmental room cues. Both *Fmr1* and WT mice performed similarly on Morris water maze reversal learning (Fig. 6, Table 5). Latency to find the hidden platform during reversal learning revealed a significant effect of day, no effect of genotype, and no interaction (Fig. 6*A*). Distance traveled similarly showed a significant effect of day, no effect of genotype, and no interaction (Fig. 6*B*). Swim speed showed no effect of day, no effect of genotype, and no interaction (Fig. 6*C*). Probe trial performance 3 h after reversal training on day 4 revealed significant quadrant preference (Fig. 6*D*) and selective target search (Fig. 6*E*) for both WT and *Fmr1* mice.

**Table 4:** Statistical results for Morris water maze (MWM) acquisition performance in *Fmr1* and WT mice

Effect	Data structure	Type of test	Power	df (between)	df (within)	*F*	*P*
MWM acquisition latency-genotype	Sphericity violated	Mixed-model ANOVA	0.19	1	22	1.31	0.3
MWM acquisition latency-day	Sphericity violated	Mixed-model ANOVA with Greenhouse–Geisser correction	1.00	4.63	101.95	25.73	5.55E-16
MWM acquisition latency-interaction	Sphericity violated	Mixed-model ANOVA with Greenhouse–Geisser correction	0.26	4.63	101.95	0.61	0.7
MWM acquisition distance-genotype	Sphericity passed	Mixed-model ANOVA	0.12	1	22	0.62	0.4
MWM acquisition distance-day	Sphericity passed	Mixed-model ANOVA	1.00	7	154	16.26	6.66E-16
MWM acquisition distance-interaction	Sphericity passed	Mixed-model ANOVA	0.36	7	154	0.85	0.5
MWM acquisition speed-genotype	Sphericity violated	Mixed-model ANOVA	0.32	1	22	2.47	0.1
MWM acquisition speed-day	Sphericity violated	Mixed-model ANOVA with Greenhouse–Geisser correction	1.00	4.49	98.82	9.64	0.0000005
MWM acquisition speed-interaction	Sphericity violated	Mixed-model ANOVA with Greenhouse–Geisser correction	0.29	4.49	98.82	0.69	0.6
WT quadrant time	Sphericity passed	Repeated-measures ANOVA with *post hoc* Dunnett’s test	0.97	3	33	7.19	0.0008
*FMR1* quadrant time	Sphericity violated	Repeated-measures ANOVA with Greenhouse–Geisser correction and *post hoc* Dunnett’s test	1.00	1.65	18.16	25.04	0.00001
WT platform crossings	Sphericity passed	Repeated-measures ANOVA with *post hoc* Dunnett’s test	0.99	3	33	12.54	0.00001
*FMR1* platform crossings	Sphericity violated	Repeated-measures ANOVA with Greenhouse–Geisser correction and *post hoc* Dunnett’s test	1.00	1.36	14.98	28.16	0.00003

**Table 5:** Statistical results for Morris water maze reversal performance in *Fmr1* and WT mice

Effect	Data structure	Type of test	Power	df (between)	df (within)	*F*	*p*
Latency reversal-genotype	Sphericity violated	Mixed-model ANOVA	0.05	1	22	0.001	0.97
Latency reversal-day	Sphericity violated	Mixed-model ANOVA with Greenhouse–Geisser correction	1.00	2.16	47.58	10.12	0.0002
Latency reversal-interaction	Sphericity violated	Mixed-model ANOVA with Greenhouse–Geisser correction	0.22	2.16	47.58	0.82	0.5
Distance reversal-genotype	Sphericity violated	Mixed-model ANOVA	0.07	1	22	0.16	0.7
Distance reversal-day	Sphericity violated	Mixed-model ANOVA with Greenhouse–Geisser correction	0.99	2.17	47.66	7.91	0.0008
Distance reversal-interaction	Sphericity violated	Mixed-model ANOVA with Greenhouse–Geisser correction	0.37	2.17	47.66	1.46	0.2
Speed reversal-genotype	Sphericity passed	Mixed-model ANOVA	0.05	1	22	0.04	0.8
Speed reversal-day	Sphericity passed	Mixed-model ANOVA	0.41	3	66	1.63	0.2
Speed reversal-interaction	Sphericity passed	Mixed-model ANOVA	0.33	3	66	1.28	0.3
WT reversal quadrant time	Sphericity violated	Repeated-measures ANOVA with Greenhouse–Geisser correction and *post hoc* Dunnett’s test	0.80	1.94	21.37	4.08	0.3
*FMR1* reversal quadrant time	Sphericity violated	Repeated-measures ANOVA with Greenhouse–Geisser correction and *post hoc* Dunnett’s test	1.00	2.02	22.24	20.84	0.000008
WT reversal platform crossings	Sphericity passed	Repeated-measures ANOVA with *post hoc* Dunnett’s test	0.91	3	33	5.41	0.004
*FMR1* reversal platform crossings	Sphericity violated	Repeated-measures ANOVA with Greenhouse–Geisser correction and *post hoc* Dunnett’s test	1.00	1.97	21.68	9.91	0.0009

## Discussion

The present studies attempted to challenge the cognitive capabilities of *Fmr1* mice by implementing the following four touchscreen tasks: visual discrimination, reversal of the visual discrimination, nonmatching to position, and delay-dependent nonmatching to position. All revealed normal performance in *Fmr1* mice. In the present studies, normal performance was found in *Fmr1* mice on touchscreen pairwise discrimination learning and reversal. Both days and trials to criterion were similar between genotypes, indicating that there were no motivational differences between genotypes. Importantly, Dickson et al. (2013) reported differences between *Fmr1* and WT mice in a serial pairwise discrimination reversal task. However, these differences were selectively observed when a specific stimulus pair was used (+ or X), but not during the reciprocal pairing (X or +), and this effect was limited to reversal 2. Interestingly, a significant bias for X over + was seen on the first day of acquisition in both genotypes. In the present studies using X and = as the stimulus pairs, the specific stimulus rewarded during the first day of acquisition did not show a bias in our laboratory (unpublished data). As we did not conduct serial reversal, it is unclear whether an initial symbol bias could contribute to an *Fmr1* deficit.

No differences in trials to criterion were observed in initial nonmatch rule learning or initial delay acquisition, which indicates equal motivation between *Fmr1* and WT mice, similar to what was observed during touchscreen pairwise visual discrimination learning. A comparison of WT and *Fmr1* performance at the individual delays across the 25 d of testing under the complete delay schedule (1, 3, and 10 s) revealed no genotype differences at any delay. Performance at 1 s reveals very high performance (∼90% correct) in both genotypes when working memory was virtually untaxed. Performance at 3 s was significantly worse than 1 s performance (∼75% correct), suggesting that these task parameters were sufficiently challenging to test moderate working memory load in these mice. Performance at 10 s was near chance (50% correct) in both genotypes, indicating that 10 s is sufficient to produce a floor effect in this task. Furthermore, while touchscreen testing in rats on nonmatch tasks has successfully used trial-unique delayed nonmatching-to-location (TUNL) to more completely prevent mediating strategies (Talpos et al., 2010), this strategy has only recently been successfully applied to mice after substantial modifications (Kim et al., 2015). The TUNL task in mice has the potential limitation of the subject using mediating strategies. Locations of the sample in positions in the center of the array are inherently more difficult than sample locations at sides of the array (Kim et al., 2015). While mediating strategies are possible (i.e., orienting body position toward target location) when non-trial-unique choice locations are used, these were not observed in the present study. The 10 s delays were sufficient to produce chance performance, which would not be the case if mediating strategies were being used. Further, asymptotic performance was sustained over the course of 25 d of final delay schedule testing, which also suggests a lack of mediating strategies.

The present studies also compared performance of the FVB/AntJ background strain in the delayed nonmatching to position task to performance of the C57BL/6J inbred strain in order to determine the baseline capabilities of the *Fmr1* background strain. Interestingly, reaching criterion on the initial delays of 1 and 3 s took significantly longer in the FVB/AntJ strain than in B6 mice. Further, once the complete delay schedule was implemented (1, 3, and 10 s), the FVB mice performed significantly worse than B6 mice at 3 s, demonstrating the sensitivity of our methods to detect performance deficits. Results with the inbred strains confirmed that a delay of 3 s was sufficient to detect performance deficits, as the performances of inbred strains, *Fmr1*, and WT were all well above chance levels.

Given the variable literature on *Fmr1* mouse performance on Morris water maze spatial learning and memory (The Dutch-Belgian Fragile X Consortium, 1994; Kooy et al., 1996; D'Hooge et al., 1997; Paradee et al., 1999; Yan et al., 2004; Baker et al., 2010; Uutela et al., 2012; Tian et al., 2015), we conducted this task using methods that detected deficits in other lines of mutant mice (Holmes et al., 2001; Rustay et al., 2005; Brielmaier et al., 2012), with slight modifications to make the task more difficult. No genotype differences between *Fmr1* and WT mice were observed on acquisition, probe trial, reversal, or reversal probe trial. Swim speed was similar between genotypes during acquisition and reversal learning, indicating intact motor abilities. Interestingly, with one exception (Baker et al., 2010), deficits that were previously observed in *Fmr1* mice during water maze acquisition were not found in probe trial performance (The Dutch-Belgian Fragile X Consortium, 1994; Kooy et al., 1996; D'Hooge et al., 1997; Paradee et al., 1999; Uutela et al., 2012), indicating the uniform capability to use distal spatial cues to navigate toward a hidden platform. Further, most of the water maze reports used the B6 background (The Dutch-Belgian Fragile X Consortium, 1994; Kooy et al., 1996; D'Hooge et al., 1997; Paradee et al., 1999; Uutela et al., 2012), avoiding the potential concern of retinal degeneration in the FVB/NJ background. While there are some reports of background strain-dependent phenotypes in the *Fmr1* mouse (Spencer et al., 2011), a recent review of the effect of background strain on cognitive abilities in *Fmr1* mice did not reveal consistency in strain-specific cognitive deficits (Kazdoba et al., 2016). While we cannot exclude that there might be water maze conditions that would reveal a deficit in this task, such as a larger pool size or colder water, our standard testing conditions did not reveal a deficit, as would be expected from a strong mouse model of FXS.

Since the original generation of the *Fmr1* knock-out mouse model of fragile X syndrome in 1994, hundreds of publications have evaluated the behavioral phenotypes of *Fmr1* mice, on both B6 and FVB genetic backgrounds. In most cases, normal performance on learning and memory tasks was apparent in well validated and established gold standard mouse cognitive assays; however, these findings varied considerably. Some groups showed deficits in passive avoidance (Qin et al., 2002; Dölen et al., 2007; Yuskaitis et al., 2010; Veeraragavan et al., 2011a; Ding et al., 2014; Michalon et al., 2014), while others did not (The Dutch-Belgian Fragile X Consortium, 1994; Veeraragavan et al., 2011b, 2012). Deficits in contextual, cued, and/or trace-cued fear conditioning were reported by some groups (Paradee et al., 1999; Zhao et al., 2005; Auerbach et al., 2011; Ding et al., 2014), while other researchers failed to detect fear conditioning deficits (Dobkin et al., 2000; Peier et al., 2000; Van Dam et al., 2000; Baker et al., 2010; Uutela et al., 2012). Morris water maze acquisition and reversal were impaired in *Fmr1* mice in some studies (The Dutch-Belgian Fragile X Consortium, 1994; Kooy et al., 1996; D'Hooge et al., 1997; Baker et al., 2010; Tian et al., 2015), while not in others (Paradee et al., 1999; Yan et al., 2004; Uutela et al., 2012). It is possible that the small dimensions of the specific apparatus used here (120 cm) contributed to the lack of observed phenotype. Novel object recognition and object location memory were detected in multiple reports (Ventura et al., 2004; Busquets-Garcia et al., 2013; King and Jope, 2013; Seese et al., 2014), but not in all (Yan et al., 2004). Five-choice serial reaction time has shown both deficiencies and normal performance in *Fmr1* mice (Moon et al., 2006; Krueger et al., 2011; Kramvis et al., 2013; Sidorov et al., 2014). As mentioned previously, background strain differences have been reported in *Fmr1* mice in some tasks (Paradee et al., 1999; Dobkin et al., 2000; Spencer et al., 2011); however, cognitive testing has not revealed a consistent background strain-dependent phenotype. Very large group sizes used in some of the cited publications (The Dutch-Belgian Fragile X Consortium, 1994; Kooy et al., 1996; D'Hooge et al., 1997; Baker et al., 2010) may have been needed to detect subtle cognitive deficits in standard learning and memory paradigms. Because of these diverse findings, we sought to develop more sensitive touchscreen tasks to detect robust cognitive deficits in *Fmr1* mice on the FVB/AntJ background, which could be used in preclinical discovery of therapeutics.

In conclusion, touchscreen tasks for mouse models of neurodevelopmental disorders with intellectual disabilities offer advantages in designing tasks that allow the researcher to titrate the demands on working memory and evaluate various cognitive domains, using equipment similar to that used in human subjects with intellectual disabilities (Green et al., 2009; Van der Molen et al., 2010; van Nieuwpoort et al., 2011; Berry-Kravis et al., 2013; Díez-Juan et al., 2014). Our findings with a new touchscreen DNMTP task revealed that *Fmr1* mutant mice on the FVB/AntJ background performed as well as their WT controls on a delay-dependent working memory task. Normal performance by *Fmr1* mice on a variety of touchscreen and other types of learning tasks confirms rather than resolves the conundrum that the *Fmr1* mouse model does not recapitulate the cognitive profile of human FXS, at least on this array of behavioral tasks. It remains possible that our tasks, while designed to challenge working memory capacity, may have been insufficiently difficult to reveal cognitive deficits in *Fmr1* mice. Unfortunately, the present findings confirmed the general lack of significant cognitive phenotypes in the Fmr1 mouse model of FXS. The lack of robust cognitive phenotypes, even on a challenging working memory task, is an important contribution because it suggests that the *Fmr1* mouse model may not be as useful as originally predicted.

## Synthesis

The decision was a result of the Reviewing Editor Howard Eichenbaum and the peer reviewers coming together and discussing their recommendations until a consensus was reached. A fact-based synthesis statement explaining their decision and outlining what is needed to prepare a revision is listed below. The following reviewers agreed to reveal their identity: Tim Bussey, Emma Wood

The manuscript provides clear cut and new data - a lack of impairment by FMR1 ko FVB/AntJ mouse model of Fragile X syndrome on a suite of 4 touchscreen tasks assessing visual discrimation, reversal, nonmatching to sample, and delayed non-matching to sample. This adds to a large body of behavioural data on the fmr1 ko mouse in which either no cognitive impairments, or relatively mild or inconsistent (across strains and testing protocols) impairments have been reported.

However, it is not clear how the new findings advance the field, as they have neither resolved any of the previous conflicts, nor provided an explanation for the lack of impairment observed. They have also not shown definitely that these animals are not cognitively impaired - simply that their performance is as good as wt controls (i.e. they are not impaired) on these specific tasks. The significance of the findings needs to be further discussed. More detailed comments by the reviewers follows:

1. The authors should expand on why the negative findings are important. One reason is that the mouse model they use has construct validity in that the knock-out results in reduction in the protein as intended. It has been regarded some as a useful model. The behavioural results from this mouse have been weak and/or inconsistent. If the mouse is not a good model, then we need to know this definitively so people stop wasting their time with it and do more productive science. This study provides good evidence that we should try a different approach.

2. Also the authors should expand on how the negative findings are convincing. The one result worth discussing in this regard is from the delayed nonmatch-to-sample task. First, the 10 second delay data are not useful because all animals are at chance level performance (so no room for an impairment to emerge). (I am not suggesting these be removed; they are important from the point of view of understanding the parameters of the task.) But there is also no hint of an impairment at 3 seconds. The question is, have the mice been brought down sufficiently from ceiling. This can be subjective, but they drop down to about 70% and I think if these animals had a problem with working memory it would have at least started to come out at this delay. Furthermore, the authors include a strain comparison which demonstrates that differences between genotypes can indeed be observed using this task, and at 3 seconds as well, thus showing the task had adequate sensitivity to detect differences.

3. The authors should expand on why there have been inconsistencies in the previous literature, for example by comparing the effects of knocking out the FMR1 gene across strains. Given the strain difference in the performance of the wild type mice on the DMP task, it is curious that the possibility of a strain-dependent ko effect was not also investigated. Also, it could be argued that the touchscreen tasks used in the current study may not have been challenging enough to reveal a deficit, and/or did not tap the precise cognitive abilities that are affected by disrupting FMR1 function.

Minor comments:

1. Page3 line 38 "reduced" The model is not a knock down, is a knock out model, so the FMRP is not reduced, it is completely absent.

2. Page3 line 53 "Visual based touch sensitive technology in operant chambers are" is

3. Page4 line 57 "outstanding" I think this comment is unnecessary, and in general, there appears to be something of a sales pitch for the touchscreen tasks which is not really warranted - the published papers and data can speak for themselves.

4. Page 4 line 63 "traditionally" that's a bit of an overstatement.

5. Page 5 row 78 "(2) identify cognitive deficits in tasks that include the capacity to increase demands on working memory; (3) employ tasks that require distinct and overlapping neural circuitry": This sentence is overplaying the advantage of the touchscreen tasks - while these tasks may well be able to accomplish this, this can also be done by non touchscreen, and even non-operant tasks.

6. The tasks which have been used in humans resemble the mouse tasks but it would be good to include a clear statement on how subjects with FXS perform on visual discrimination, reversal, and delayed nonmatch to position tasks (plus delays) analogous to those used in the current study. This may provide a clearer rationale by which these particular tasks were used in the current mouse study.

7. Page 5 line 83, a distinction is made between cortically mediated working memory vs hippocampally mediated spatial navigation in a water maze. The implication is (I think) that the touchscreen DMP task does not require intact hippocampal function - has this been shown? It is certainly the case that many forms of DMP are hippocampus dependent.

8. Page 5 in subjects paragraph. Include a statement about genotyping. Were the animals tested with experimenters blind to genotype? How did they counterbalance conditions in their experiments?

9. Page 7 A diagram with the different phases of the experiment (for the touchscreen tasks) would be useful, because there are so many stages it becomes complicated.

10. General comment on the water maze. The fact that they didn't see a difference between groups could be because of the small dimensions of the pool, the size of the platform, the fact that they didn't interleave the mice between trials etc. So a null result in this task using this background strain doesn't really add anything to the field. Furthermore the strain in which a mild deficit in the reversal stage of the task has most often been observed is the Bl6 strain, so the current result does not conflict with that.

11. Page 15 lines 306-311. I don't really understand the advantage of using a mouse strain which has worse performance to start with. On top of this the statement in line 310 is false, as they didn't test Fmr1 ko mice in both strains to see which is best.

12. Page 17 line362 another reference for object recognition Busquets-Garcia et al 2013 Nat medicine.

13. Page18 line384-5 How did they counterbalance? Didn't some animals get X and = and some others = and X ?

14. General comment on the figures. Add keys with the different symbols directly on the figures rather than include them in the legend.

15. Stats for the delayed nonmatching to place task delay phase (data in figure 2A and B, and also figure 3A and 3B) do not directly compare between the groups of mice. Rather, they compare between delays within each group. Why not do a mixed model ANOVA with group as a between subjects factor?

16. The version of the task the authors use uses two locations on the screen. My understanding is that the touchscreen nonmatching-to-sample task usually uses many more locations in order to avoid mediating strategies to minimize the requirement for memory, which has been shown several times to occur in the two-lever version of the task (mediating strategies are mentioned by the current authors in the Results section). Indeed, other authors have shown that such strategies do not exist in the multi-location touchscreen versions. Can the authors provide some evidence that a deficit was not masked by such mediating strategies in their mice in their two-location task, even if only anecdotal?

17. "Pairwise visual discrimination relies on the perirhinal cortex (Winters et al., 2010)." That study used difficult to discriminate stimuli. Work from the Aggleton lab suggests simple pairs such as those used in the present study do not require perirhinal cortex.
